# Survival rate of patients with combined hepatocellular cholangiocarcinoma receiving medical cannabis treatment: A retrospective, cohort comparative study

**DOI:** 10.12688/f1000research.123250.2

**Published:** 2025-04-09

**Authors:** Narisara Phansila, Paopong Pansila, Adisorn Wongkongdech, Niruwan Turnbull, Mahalul Azam, Ranee Wongkongdech

**Affiliations:** 1 Chiang Kwan Hospital, Roi-et Province, 45000, Thailand; 2 Faculty of Medicine, Mahasarakham University, Mahasarakham, 44000, Thailand; 3 Public Health and Environmental Policy in Southeast Asia Research Cluster (PHEP SEA Thailand), Mahasarakham University, Mahasarakham, 44150, Thailand; 4 Faculty of Public Health, Mahasarakham University, Mahasarakham, Mahasarakham Province, 44150, Thailand; 5 Public Health Department, Faculty of Sport Sciences, Universitas Negeri Semarang, Semarang, 50229, Indonesia

**Keywords:** Survival rate, medicinal cannabis, combined hepatocellular cholangiocarcinoma, cHCC-CC, palliative care, Northeastern Thailand

## Abstract

**Background:** Cholangiocarcinoma (CCA) incidence in Northeastern Thailand is very high and a major cause of mortality. CCA patients typically have a poor prognosis and short-term survival rate due to late-stage diagnosis. Thailand is the first Southeast Asian country to approve medicinal cannabis treatment, especially for palliative care with advanced cancer patients.

**Methods:** A retrospective cohort comparative study of survival rates among 491 newly diagnosed advanced CCA patients was carried out between September 1, 2019, and June 30, 2021. A total of 404 patients were in the standard palliative care pain management treatment group (ST), and 87 were in the medicinal cannabis treatment group (CT). Patients with CCA were recruited from four tertiary hospitals and two secondary hospitals in five provinces of Northeast Thailand. The cumulative survival rates were calculated by the Kaplan-Meier method, and independent prognostic factors were investigated using Cox regression.

**Results**: For ST patients, there was a total follow-up time of 790 person-months, with a mortality rate of 48.35/100 person-months. For CT patients the total follow-up time was 476 person-months, with mortality rate of 10.9/ 100 person-months. The median survival time after registration at a palliative clinic was 0.83 months (95% CI: 0.71–0.95) for ST and 5.66 months (95% CI: 1.94–9.38) for CT. Multivariate analysis showed that CT treatment protocol was associated with a significantly better survival (P value <0.001; median time of CT, 5.66 months (95% CI: 1.94–9.38); median time of ST, 0.83 months (95% CI: 0.71–0.95). Therefore, CT had a reduced probability of dying from the disease (HR _adj_., 0.28 (95% CI: 0.20–0.37)

**Conclusions**: The medical cannabis increased overall survival rates among CCA patients.

## Introduction

Combined hepatocellular cholangiocarcinoma (cHCC-CC) is a rare, but severely aggressive primary liver cancer manifesting characteristics of both hepatocellular carcinoma (HCC) and cholangiocarcinoma (CC). The incidence rate is approximately 0.59 per 1,000,000 populations worldwide ^1^ but it is highly prevalent in Thailand. ^2^ The highest reported CC incidence internationally is in northeastern Thailand, 118.5 per 100,000, in Khon Kaen Province, which is over 100 times higher than the global rate. ^3^

CC is generally asymptomatic in early stages and is usually diagnosed late when the disease has already metastasized. Late-stage diagnosis limits the effective therapeutic options and has an aggressive disease course ^4^ and very poor prognosis, ^5^ resulting in lower survival rates. Previous studies have shown the median post-diagnosis survival of CC patients to be about 9 months (95% CI: 7–11), with 1-, 3-, and 5-year survival rates at 43.4, 21.5, and 17.1%, respectively. ^1^ Mean overall survival rate at 1-, 3-, and 5-year was 66.6, 41.5, and 32.7% for patients with transitional cHCC-CC, with median survival time from diagnosis 4.3 months (95% CI: 3.3–5.1), ^6^ and after supportive treatment was 4 months. ^7^ Survival time was increased among CC patients receiving surgery (an average of 29.38 months), best supportive treatment was 5.12 months and 13.38 months for chemotherapy patients. ^8^

At present, medical cannabis products are in use in many countries. ^9^ Cannabis as a palliative treatment for patients with cancer appears to be well-tolerated, effective and a safe pain-relief option with significant improvement in quality of life shown after 6 months of treatment. ^10^ In patients with cancer, cannabinoids have mainly been used as part of palliative care to alleviate pain, relieve nausea and stimulate appetite. ^11^ Thailand legalized medical cannabis in February 2019, becoming the first country in Southeast Asia to regulate medical treatment. ^12^ Currently, there are two treatment options for palliative cancer patients in Thailand; the standard current treatment and the new cannabis treatment. However, to the best of our knowledge, no studies on the survival rate of patients treated with medicinal cannabis from the patients’ perspective have been carried out to date. The present study aims to compare survival rates in palliative cHCC-CC patients who were treated with standard treatment (ST) or cannabis treatment (CT) palliative care protocols.

## Methods

### Ethical approval

This study was reviewed and approved by the Mahasarakham University Human Research Ethics Committee (Reference No. 204/2563; approved on July 24, 2020), and Roi-Et Regional Hospital (Reference RE064/2563; approved on August 26, 2020), Buriram Regional Hospital Ethics Committee for Human Research, based on the Declaration of Helsinki and the ICH Good Clinical Practice Guidelines (Reference No. GCP0066/2563; approved on February 4, 2020). Because of its retrospective manner, informed consent was waived by the Roi-Et Regional Hospital and Buriram Regional Hospital. Data were collected from August 30, 2020, to June 30, 2021, which collected event data on 491 cases from September 1, 2019, to June 30, 2021.

### Study design

An observational analytical study using a retrospective cohort design was conducted with 491 cHCC-CC patients (404 received ST and 87 received CT), diagnosed at least by ultrasonography and treated with supportive care at palliative care and/or cannabis care clinics between September 1, 2019, and June 30, 2021. Data were extracted from four tertiary hospitals and two secondary hospitals in five provinces of northeastern Thailand (Roi-Et Regional Hospital, Buriram Regional Hospital, Surin Provincial Hospital, Sawang Dandin Crown Prince Hospital, Panna Nikhom Hospital, and Pana Hospital). Follow-up was conducted until the outcome was reached or the study concluded, with additional insights gathered through interviews with oncologists from eight hospitals regarding treatment protocols.

### Data collection

**Figure 1.**
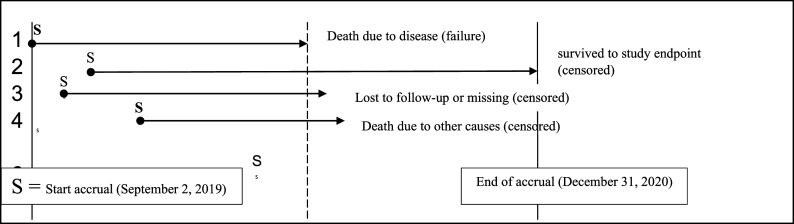
Study flow diagram for participant accrual and outcomes.

Patients were eligible for inclusion if they were newly diagnosed with cholangiocarcinoma (CCA) or hepatocellular carcinoma (HCC) between September 2019 and December 2020, were over the age of 18, and registered at either the palliative or cannabis clinic. Exclusion criteria included prior cannabis use before study registration or incomplete medical records. Participants were followed from registration until death or the study endpoint (June 30, 2021). Follow-up was conducted through clinic visits, medical record reviews, and linkage to the national death registry. Data on survival time, treatment response, and adverse events were collected at each visit. Censored data were recorded for participants who were still alive at the end of the study or lost to follow-up. Follow-up status was verified through medical records, the national death registry, and telephone calls to the community health centers’ patient or public health officers ( Figure 1).

In this study, we examined the survival outcomes of patients with CCA and HCC based on various factors, excluding pain level as a variable. While pain level was not included in the analysis, we acknowledge this as a limitation and have clarified it here.

### Independent and dependent variables

The independent variables included age at registration (Palliative clinic and/or Cannabis clinic), gender, cancer treatment, and the period from diagnosis to registration. The dependent variable was the post-diagnosis survival time of patients with CCA and HCC. To calculate survival time, the starting point was identified as the registration date, and the follow-up period ended when a patient died or the study was completed.

### Statistical methods

Statistical analysis was performed with Stata (RRID:SCR_012763) version 15 (free alternative, Rstudio). Descriptive statistics were used to present baseline characteristics and clinical subject data. Frequency and percentages were constructed to describe categorical data and expressed as the means deviation (in SD) or medians with ranges to describe continuous data. The Kaplan-Meier method was used for observing survival duration with 95% confidence intervals (95% CI). Then between-group comparisons were evaluated using a log-rank test. The test for associations between the diverse covariates and survival rate was performed using the Cox proportional hazard regression model. The results were submitted as hazard ratios (HR) with 95% CI for HR. A p-value less than 0.05 is typically considered to be statistically significant.

## Results

### Standard medical treatment

Diagnosis and assessment are conducted using basic diagnostic methods such as endoscopy, CT/MRI scans, and blood tests. Standard medical treatments include chemotherapy (CT), surgery, and palliative care approaches. Follow-up involves monitoring the patient’s progress through physical examinations and laboratory test results to assess the effectiveness of the treatment.

### Medical cannabis treatment

Patients must provide diagnostic results confirming advanced-stage cancer, such as biopsy findings or CT/MRI scans showing metastasis. Treatment involves prescribing cannabis products, including THC:CBD 1:1, THC, and CBD cannabis oil. Follow-up care includes adjusting the treatment based on the patient’s response, with regular check-ups to monitor progress and make necessary adjustments to the cannabis treatment plan.

### Participants’ Characteristics and Survival Rates of Patients with CCA/HCC Treated with Cannabis Therapy (CT) and Standard Therapy (ST)

Table 1 shows the characteristics of the study participants. ^15^^,^^16^ There were 491 patients (296 male subjects and 195 female subjects) with cHCC-CC; there were 404 in the ST group (242 male subjects and 162 female subjects) and 87 in the CT group (54 male participants and 33 female participants). The mean age of those in the ST group was 66.60 years old, and the mean age of the CT group was 65.64 years old. Most patients (43.38%) were 70 years of age. More than 71.53% in the ST received cancer chemotherapy and combinations, and 49.42% of the CT group also received palliative care. Mean point of diagnosis with advanced cHCC-CC to registration was 6.12 months for ST, and 5.46 months for CT. Most patients (38.49%) were registered at the palliative and/or cannabis care clinic, and 94.60% (ST) and 59.80% (CT) had passed away by the end of the study. The total follow-up time for ST patients was 790 person-months, with a mortality rate of 48.35/100 person-months. For the CT group follow-up was 476 person-months, with a mortality rate of 10.9./100 person-months for CT.

**Table 1.** Baseline characteristics of included patients (n=491). ST, standard palliative care pain management treatment group; CT, medicinal cannabis treatment group.

Variable	Patient treatment group		Median time, month (95% CI)			Person-time, month		Incidence rate/ 100 person/month		HR _adj._ 95% CI	P-value
	ST (n=404, %)	CT (n=87, %)	ST (n=404, %)	CT (n=87, %)	P-value	ST	CT	ST	CT		
**Overall survival rate**	0.83 (0.71–0.95)	5.66 (1.94–9.38)			<0.001						
**Age, years, mean (SD)**	66.60 (11.67)	65.64 (9.82)			<0.001						
<60	105 (25.99)	24 (27.59)	0.83 (0.60–1.00)	5.67 (2.87–15.00)		170	147	0.59	0.08	1	0.212
60–69	121 (29.95)	28 (32.18)	0.93 (0.73–1.04)	3.27 (2.0–12.00)		244	128	0.47	0.13	0.85 (0.66–1.09)	
≥70	178 (44.06)	35 (40.23)	0.83 (0.67–1.27)	6.00 (2.33–10.03)		375	200	0.44	0.11	0.87 (0.68–1.09)	
**Sex**											
Male	242 (81.8)	54 (18.2)	0.73 (0.67–0.93)	6.00 (3.07–10.03)	<0.001	427	300	0.53	0.10	1	0.236
Female	162 (83.1)	33 (16.9)	0.97 (0.83–1.20)	3.50 (1.77–9.50)		362	175	0.42	0.11	0.89 (0.73–1.08)	
**Cancer treatment**											
Surgery	28 (6.93)	4 (4.59)	1.33 (0.30–2.50)	2.00 (1.83–10.00)	<0.001	106	14	0.20	0.21	1	0.106
Chemotherapy	140 (34.65)	18 (20.70)	0.93 (0.73–1.0)	9.50 (5.17–15.00)		209	139	0.65	0.06	1.43 (0.93–2.2)	
Combine	149 (36.88)	22 (25.29)	0.83 (0.67–1.27)	7.00 (1.67–15.00)		311	121	0.45	0.09	1.27 (0.82–1.93)	
Palliative care	87 (21.54)	43 (49.42)	0.73 (0.5–0.93)	3.07 (2.17–.8.33)		162	201	0.51	0.14	1.23 (0.79–1.92)	
**Treatment protocol**											
ST	404	87	0.83 (0.71–0.95)		<0.001					1	<0.001
CT	(82.3)	(17.7)	5.66 (1.94–9.38)							0.28 (0.20–0.37)	
**Period advanced diagnosis to register**											
Mean (SD)	6.12 (2.55)	5.46 (2.94)			<0.001						
< 3 months	60 (85.14)	40 (45.98)	0.93 (0.67–2.00)	3.17 (2.17–9.00)		115	115	0.54	0.14	1	0.844
3–6 months	204 (49.50)	22 (25.28)	0.83 (0.67–0.97)	8.17 (2.87–15.00)		406	406	0.46	0.08	1.31 (1.01–1.71)	
6–9 months	94 (27.23)	8 (9.20)	1.07 (0.67–1.67)	5.00 (0.73–8.00)		210	210	0.41	0.09	1.21 (0.89–1.65)	
>9 months	46 (39.11)	17 (19.54)	0.67 (0.44–1.77)	5.17 (200–9.00)		59	59	0.72	0.09	1.16 (0.82–1.63)	
**Status at the end of study**											
Passed away	382 (94.60)	52 (59.80)									

The survival rate data after registration at either the palliative or cannabis care clinic. The cumulative 3, 6, 9 and 12 months survival rates were 28.80% (95% CI: 24.72–32.99), 20.00% (95% CI: 16.35–23.92), 16.50% (95% CI: 12.86–20.55) and 15.75% (95% CI: 12.04–19.92) for ST, 60.48% (95% CI: 49.35–69.91), 48.63% (95% CI: 36.78–57.70), 35.73% (95% CI: 23.83–47.74) and 29.98% (95% CI: 18.15–42.73) for CT, respectively. The median duration of survival was 0.83 months (95% CI: 0.71–0.95) for ST and 5.66 months (95% CI: 1.94–9.38) for CT. None of the demographic factors were significantly associated with survival time for either ST or CT. Comparing ST with CT, there was a statistically significant difference in age, sex, cancer treatment and period diagnosis with advanced cHCC-CC to register factors (p-value<0.05). There were factors found that affected the survival of patients receiving palliative care for liver and bile duct cancer. The most significant treatment factor found was between those patients who received standard therapy and those who received medical cannabis. Those on standard therapy were 3.57% more at risk of death than those on cannabis.

Multivariate analysis showed that CT treatment protocol was associated with improved patient survival, which was statistically significant (P value <0.001, the median time of CT, 5.66 months (95% CI: 1.94–9.38) and ST, 0.83 months (95% CI: 0.71–0.95), HR _adj_, 0.28 (95% CI: 0.20–0.37).

## Discussion

The impact of two types of treatment that affect the survival of cHCC-CC patients who either had supportive treatment at palliative clinic or a cannabis clinic. CT was the most effective treatment, with an overall survival time of 5.66 months, while overall survival time was 0.83 months for ST. Meanwhile, the overall survival times are consistent with other findings for after supportive treatment ^7^ where survival time was only 4.3 months post-diagnosis. Patients diagnosed at an advanced stage were twice as likely to pass away (HR: 1.8, 95% CI: 1.1–2.9). ^13^ By contrast, patients with advanced cancer using cannabis showed a significantly decreased overall survival compared to non-users. ^14^

In the univariate analysis, cancer treatment and period of diagnosis with advanced cHCC-CC to registration were associated with survival rate. It was found that the ST registered patients survived less than three months after being diagnosed with advanced-stage cHCC-CC. The reason for this might be that some patients had been consulting and were being cared for by an oncologist or other doctor rather than those patients who were registered for and receiving supportive treatment at a Palliative Clinic. Furthermore, most patients had been treated with a combination of surgery and chemotherapy, before being admitted to a Palliative Clinic. Although the registered patients at the Cannabis Clinic were >70 years old, they had no cancer treatment, only supportive treatment at the Cannabis Clinic. At the community hospitals where CT/MRI/biopsy/US have shown advanced organ metastases others who received treatment at a Cannabis clinic without waiting for a consultation with an oncologist were able to receive chemotherapy along with cannabis. This study has several limitations. A number of patients in the CT group dropped out before completion of the study. As a consequence, most patients suffering from advanced cancers and receiving heavy oncological treatments were older adults.

Patients with CCA have poor prognosis and short-term survival at the time of diagnosis. Registration and decision-making at the standard and/or cannabis clinic in each hospital differs across physicians, patients, families, stages of disease, organ metastasis, methods of treatment, and severity of symptoms. To the best of our knowledge, this is the first study that has compared survival rate and quality of life of CHCA/CCA patients who received either ST or CT across tertiary and secondary hospitals and across five provinces. Medical cannabis used in this study were standardized cannabis preparations made by the Thailand Food and Drug Administration. The side effects, safety, benefits and harms of the cannabis produced have been reviewed and are considered appropriate patient treatment. Prescribing doctors are trained, registered prescribers of medical cannabis.

## Author contributions

N.P., contributed to Conceptualization, Data Curation, Formal Analysis, Resources, Methodology, Investigation, Writing – Original Draft. P.P., and A.W., contributed to Methodology, Investigation, Resources, Validation, Formal Analysis, Visualization. N.T., contributed to Conceptualization, Investigation, Supervision, Visualization, Writing – Review & Editing. M.A., contributed to Investigation, Visualization, Writing – Review & Editing. R.W., contributed to Conceptualization, Project Administration, Methodology, Investigation, Writing – Review & Editing, Funding Acquisition, and Supervision.

## Data Availability

Figshare: Data_survival_cannabis. https://doi.org/10.6084/m9.figshare.20101193. ^15^ Figshare: F1000_survival_table1_narisara_ranee. https://doi.org/10.6084/m9.figshare.20486913. ^16^ Data are available under the terms of the Creative Commons Attribution 4.0 International license (CC-BY 4.0).

## References

[1] Wang J Li E Yang H : Combined hepatocellular-cholangiocarcinoma: a population level analysis of incidence and mortality trends. *World J. Surg. Oncol.* 2019 Dec;17(1):43. 10.1186/s12957-019-1586-8 30813932 PMC6394104

[2] Titapun A Pugkhem A Luvira V : Outcome of curative resection for perihilar cholangiocarcinoma in Northeast Thailand. *World J. Gastrointest. Oncol.* 2015 Dec 15;7(12):503–512. 10.4251/wjgo.v7.i12.503 26691730 PMC4678397

[3] Alsaleh M Leftley Z Barbera TA : Cholangiocarcinoma: a guide for the nonspecialist. *Int. J. Gen. Med.* 2019;12:13–23. 10.2147/IJGM.S186854 30588065 PMC6304240

[4] Banales JM Marin JJG Lamarca A : Cholangiocarcinoma 2020: the next horizon in mechanisms and management. *Nat. Rev. Gastroenterol. Hepatol.* 2020 Sep;17(9):557–588. 10.1038/s41575-020-0310-z 32606456 PMC7447603

[5] Loosen SH Gaisa NT Schmeding M : Prolonged Survival of a Patient with Advanced-Stage Combined Hepatocellular-Cholangiocarcinoma. *Case Rep. Gastroenterol.* 2020 Dec 10;14(3):658–67. Page 13/16. 10.1159/000511034 33442346 PMC7772835

[6] Woradet S Promthet S Songserm N : Factors affecting survival time of cholangiocarcinoma patients: a prospective study in Northeast Thailand. *Asian Pac. J. Cancer Prev.* 2013;14(3):1623–1627. 10.7314/APJCP.2013.14.3.1623 23679246

[7] Thunyaharn N Promthet S Wiangnon S : Survival of cholangiocarcinoma patients in northeastern Thailand after supportive treatment. *Asian Pac. J. Cancer Prev.* 2013;14(11):7029–7032. 10.7314/APJCP.2012.14.11.7029 24377644

[8] Chanchai C Piyasatit P Muntham D : Clinical Prognostic Factors and Treatment Outcomes for the Survival of Patients with Cholangiocarcinoma in the Eastern Region of Thailand. *Asian Pac. J. Cancer Care.* 2019 Aug 1;4(4):101–105. 10.31557/apjcc.2019.4.4.101-105

[9] Carliner H Brown QL Sarvet AL : Cannabis use, attitudes, and legal status in the U.S.: A review. *Prev. Med.* 2017 Nov;104:13–23. 10.1016/j.ypmed.2017.07.008 28705601 PMC6348863

[10] Bar-Lev Schleider L Mechoulam R Lederman V : Prospective analysis of safety and efficacy of medical cannabis in large unselected population of patients with cancer. *Eur. J. Intern. Med.* 2018;49:37–43. 10.1016/j.ejim.2018.01.023 29482741

[11] Dariš B Verboten MT Knez Ž Ferk P : Cannabinoids in cancer treatment: Therapeutic potential and legislation. *Bosn. J. Basic Med. Sci.* 2019 Feb;19(1):14–23. 1. 10.17305/bjbms.2018.3532 30172249 PMC6387667

[12] World Law Group: 2020 Global Cannabis Guide – Thailand. 2020 Aug 28 [cited 2021 April 18];12(9). Reference Source

[13] Woradet S Songserm N Promthet S : Health-Related Quality of Life and Survival of Cholangiocarcinoma Patients in Northeastern Region of Thailand. *PLoS One.* 2016;11(9):e0163448. 10.1371/journal.pone.0163448 27685448 PMC5042427

[14] Bar-Sela G Cohen I Campisi-Pinto S : Cannabis Consumption Used by Cancer Patients during Immunotherapy Correlates with Poor Clinical Outcome. *Cancers (Basel).* 2020 Aug 28 [cited 2021 May 15];12(9). 10.3390/cancers12092447 Reference Source 32872248 PMC7563978

[15] Phansila N Pansila P Wongkongdech A : Data_survival_cannabis. figshare. [Dataset].2022. 10.6084/m9.figshare.20101193.v1

[16] Phansila N : F1000_survival_table1_narisara_ranee. figshare. [Dataset].2022. 10.6084/m9.figshare.20486913.v1

