# Survival rate of patients with combined hepatocellular cholangiocarcinoma receiving medical cannabis treatment: A retrospective, cohort comparative study

**DOI:** 10.12688/f1000research.123250.3

**Published:** 2025-10-03

**Authors:** Narisara Phansila, Paopong Pansila, Adisorn Wongkongdech, Niruwan Turnbull, Mahalul Azam, Ranee Wongkongdech

**Affiliations:** 1 Chiang Kwan Hospital, Roi-et Province, 45000, Thailand; 2 Faculty of Medicine, Mahasarakham University, Mahasarakham, 44000, Thailand; 3 Faculty of Public Health, Mahasarakham University, Mahasarakham, Mahasarakham Province, 44150, Thailand; 4 Public Health and Environmental Policy in Southeast Asia Research Cluster (PHEP SEA Thailand), Mahasarakham University, Mahasarakham, 44150, Thailand; 5 Public Health Department, Faculty of Sport Sciences, Universitas Negeri Semarang, Semarang, 50229, Indonesia; 6 International and National Collaborative Network and Innovation for Community Health Development Research Unit (INCNI-CHD), Mahasarakham University, Mahasarakham, 44000, Thailand

**Keywords:** Survival rate, medicinal cannabis, combined hepatocellular cholangiocarcinoma, cHCC-CC, palliative care, Northeastern Thailand

## Abstract

**Background:**

Cholangiocarcinoma (CCA) incidence in Northeastern Thailand is very high and a major cause of mortality. CCA patients typically have a poor prognosis and short-term survival rate due to late-stage diagnosis. Thailand is the first Southeast Asian country to approve medicinal cannabis treatment, especially for palliative care with advanced cancer patients.

**Methods:**

A retrospective cohort study compared survival among 491 newly diagnosed advanced CCA patients between September 2019 and June 2021. Of these, 404 received standard palliative pain management (ST), and 87 received medicinal cannabis treatment (CT). Patients were enrolled from four tertiary hospitals and two secondary hospitals in five provinces of Northeast Thailand. Cumulative survival was calculated by the Kaplan-Meier method, and independent prognostic factors were analyzed using Cox regression.

**Results:**

For ST patients, follow-up time was 790 person-months, with a mortality rate of 48.35/100 person-months. For CT patients, follow-up time was 476 person-months, with a mortality rate of 10.9/100 person-months. The median survival time after registration at a palliative clinic was 0.83 months (95% CI: 0.71–0.95) for ST and 5.66 months (95% CI: 1.94–9.38) for CT. Multivariate analysis showed CT was significantly associated with prolonged survival (HRadj = 0.28; 95% CI: 0.20–0.37; p < 0.001).

**Conclusions:**

The medical cannabis increased overall survival rates among CCA patients. In this retrospective cohort, Medicinal cannabis treatment was associated with more prolonged survival among advanced CCA patients in Northeastern Thailand. While this association remained significant after multivariable adjustment, unmeasured or residual confounding factors may have influenced the observed outcomes. Although the association remained significant after adjustment, unmeasured or residual confounders may have influenced outcomes. Further prospective studies are warranted to confirm these findings and explore potential mechanisms.

## Introduction

Combined hepatocellular-cholangiocarcinoma (cHCC-CCA) is an uncommon and aggressive form of primary liver cancer that exhibits both hepatocytic and cholangiocytic differentiation within the same tumor. ^1^ The global incidence rate is approximately 0.59 per 1,000,000 people, ^2^ whereas Thailand reports significantly higher rates. ^3^ Notably, Northeastern Thailand—particularly Khon Kaen Province—has the highest reported incidence of CCA worldwide at 118.5 per 100,000 population, exceeding the global rate by over 100-fold. ^3^ Due to its asymptomatic nature in early stages, CCA is typically diagnosed at an advanced stage when metastasis has already occurred, resulting in limited therapeutic options, aggressive disease progression, ^4^ and poor prognosis. ^5^

Previous studies have shown the median post-diagnosis survival of CCA patients to be about 9 months (95% CI 7–11), with 1-, 3-, and 5-year survival rates at 43.4%, 21.5%, and 17.1%, respectively. ^6^ Mean overall survival rate at 1-, 3-, and 5-year was 66.6, 41.5, and 32.7% for patients with transitional HCC-CC, ^7^ with median survival time from diagnosis 4.3 months (95% CI: 3.3–5.1), ^8^ and after supportive treatment was 4 months. ^9^ Survival time was increased among CCA patients receiving surgery, an average of 29.38 months, best supportive treatment, 5.12 months, and 13.38 months for chemotherapy patients. ^10^

Cannabis-based medicinal products are now legally available in several countries ^11^ and are commonly used in palliative care to alleviate pain, reduce nausea, stimulate appetite, and improve quality of life in adults with cancer. ^12^ Observational evidence also suggests that supervised medical cannabis is generally well-tolerated and associated with quality-of-life improvements over about six months of use. ^13, 14^ Thailand legalized medical cannabis in February 2019, the first country in Southeast Asia to do so ^15, 16^ and has since integrated cannabis-based options into palliative care services as an adjunct or alternative to standard care. ^17^ In a recent retrospective cohort of advanced cholangiocarcinoma treated in northeastern Thailand, patients receiving cannabis-based treatment had a median overall survival of 5.66 months and an approximate one-year (12-month) survival of 29.98%, based on Kaplan–Meier estimates, ^18^ highlighting a potential role alongside symptom management.

Survival data for cannabis-treated cholangiocarcinoma (CCA) remain limited, but some evidence suggests potential benefits. A U.S. inpatient study found that cannabis users with CCA had significantly lower in-hospital mortality than matched non-users (OR = 0.40; 95% CI: 0.16–0.97; *p* < 0.04). ^19^ In Thailand, a prospective cohort reported improved functional status and quality of life at two and four months among CCA patients receiving cannabis-based treatment compared to standard care. ^13^ Preclinical studies also indicate anticancer effects: cannabidiol (CBD) and cannabigerol (CBG) inhibited CCA cell proliferation and migration, inducing apoptosis and autophagy, ^20^ while delta-9-tetrahydrocannabinol (THC) suppressed proliferation and promoted apoptosis via MAPK/MEK and Akt pathway inhibition. ^21^ These findings, though preliminary, highlight the need for further clinical research in advanced CCA, where current survival outcomes remain poor. ^22^

This study aims to analyze survival outcomes (SA) and identify factors associated with survival rates among patients with combined hepatocellular–cholangiocarcinoma (CHCC/CCA) diagnosed by their physicians as requiring palliative care, who then choose either cannabis treatment (CT) or standard treatment (ST; conventional medical care according to national clinical practice guidelines) after receiving information on available options. Retrospective cohort data were obtained from hospital electronic medical records (EMR) and cancer clinic databases across four tertiary and two secondary hospitals in five Northeastern provinces of Thailand. The results could provide preliminary evidence to support policy discussions and the development of evidence-based palliative care strategies under the national medicinal cannabis framework.

## Methods

### Study design and setting

A retrospective cohort study was conducted with 491 patients—404 who received standard palliative care treatment (ST) and 87 who received medicinal cannabis treatment (CT). All patients were diagnosed with advanced cholangiocarcinoma (CCA) or hepatocellular carcinoma (HCC) by at least ultrasonography and managed with supportive care at a palliative care and/or cannabis care clinic between 1 September 2019 and 31 December 2020. Data were obtained from hospital electronic medical record (EMR) systems and cancer clinic databases from four tertiary hospitals and two secondary hospitals across five provinces of Northeastern Thailand: Roi Et Regional Hospital, Buriram Regional Hospital, Surin Provincial Hospital, Sawang Dandin Crown Prince Hospital, Panna Nikhom Hospital, and Pana Hospital.

### Eligibility criteria

Patients were eligible for inclusion if they were: Newly diagnosed with CCA or HCC between September 2019 and December 2020; Aged 18 years or older; and registered at either a palliative care clinic or a cannabis clinic.

Exclusion criteria included: Prior use of medicinal cannabis before study registration and incomplete medical records.

### Variables and outcomes

Independent variables included age at registration, gender, type of cancer treatment, and the period from diagnosis to registration. The primary outcome was post-diagnosis survival time, measured from the date of registration to the date of death or the study endpoint (30th June 2021). Patients alive at the end of the study or lost to follow-up were classified as censored cases.

### Follow-up procedures

Participants were followed from the date of registration until death or the study endpoint (30th June 2021). Follow-up was conducted through review of EMR entries, clinic visit records, and linkage to the national death registry.

### Cancer stage and other clinical variables

Data on cancer stage, performance status, and pain score were not consistently available in the EMR and were therefore not included as covariates in the analysis.

### Ethical approval

The study protocol was reviewed and approved by the Maha Sarakham University Human Research Ethics Committee (Reference No. 204/2563, dated July 24, 2023), the Roi Et Regional Hospital Ethics Committee (Reference No. RE064/2563, dated 26 August 2023), and the Buriram Regional Hospital Ethics Committee (Reference No. GCP0066/2563, dated 4 Febuary 2023). Permission for data access and extraction was also obtained from hospital administrators and multidisciplinary teams at each participating hospital.

### Statistical methods

Descriptive statistics summarized patient characteristics. Categorical variables were reported as frequencies and percentages, and continuous variables as means with standard deviations (SD) or medians with interquartile ranges (IQR). Baseline differences between the standard treatment (ST) and cannabis treatment (CT) groups were assessed using chi-square or Fisher’s exact tests for categorical variables and independent t-tests for continuous variables.

Survival probabilities were estimated using the Kaplan–Meier method, with group comparisons made via the log-rank test. Independent prognostic factors were identified using Cox proportional hazards regression. The proportional hazards assumption was assessed using Schoenfeld residuals and log-minus-log plots.

A two-step approach was applied:

1. Univariable analysis identified variables associated with survival (p < 0.20).
2. Multivariable analysis included these variables to adjust for confounders and estimate adjusted hazard ratios (HR_adj) with 95% confidence intervals (CI).

Statistical significance was set at p < 0.05. Analyses were performed in Stata version 17 (StataCorp LLC, College Station, TX, USA).

## Results

### Participant characteristics

Table 1 shows the study participants’ characteristics. Overall, most baseline characteristics did not differ significantly between the Standard Treatment (ST) and Cannabis Treatment (CT) groups. Significant differences (p < 0.05) were observed for chemotherapy, combined treatment, palliative care, time from diagnosis to registration (< 3 months), and survival status.

**Table 1.** Baseline characteristics of included patients (n=491). ST, standard palliative care pain management treatment group; CT, medicinal cannabis treatment group.

Variable	Patient treatment group		Median time, month (95% CI)			Person-time, month		Incidence rate/ 100 person/month		HR _adj._ 95% CI	P-value
	ST (n=404, %)	CT (n=87, %)	ST (n=404, %)	CT (n=87, %)	P-value	ST	CT	ST	CT		
**Overall survival rate**	0.83 (0.71–0.95)	5.66 (1.94–9.38)			<0.001						
**Age, years, mean (SD)**	66.60 (11.67)	65.64 (9.82)			<0.001						
<60	105 (25.99)	24 (27.59)	0.83 (0.60–1.00)	5.67 (2.87–15.00)		170	147	0.59	0.08	1	0.212
60–69	121 (29.95)	28 (32.18)	0.93 (0.73–1.04)	3.27 (2.0–12.00)		244	128	0.47	0.13	0.85 (0.66–1.09)	
≥70	178 (44.06)	35 (40.23)	0.83 (0.67–1.27)	6.00 (2.33–10.03)		375	200	0.44	0.11	0.87 (0.68–1.09)	
**Sex**											
Male	242 (81.8)	54 (18.2)	0.73 (0.67–0.93)	6.00 (3.07–10.03)	<0.001	427	300	0.53	0.10	1	0.236
Female	162 (83.1)	33 (16.9)	0.97 (0.83–1.20)	3.50 (1.77–9.50)		362	175	0.42	0.11	0.89 (0.73–1.08)	
**Cancer treatment**											
Surgery	28 (6.93)	4 (4.59)	1.33 (0.30–2.50)	2.00 (1.83–10.00)	<0.001	106	14	0.20	0.21	1	0.106
Chemotherapy	140 (34.65)	18 (20.70)	0.93 (0.73–1.0)	9.50 (5.17–15.00)		209	139	0.65	0.06	1.43 (0.93–2.2)	
Combine	149 (36.88)	22 (25.29)	0.83 (0.67–1.27)	7.00 (1.67–15.00)		311	121	0.45	0.09	1.27 (0.82–1.93)	
Palliative care	87 (21.54)	43 (49.42)	0.73 (0.5–0.93)	3.07 (2.17–.8.33)		162	201	0.51	0.14	1.23 (0.79–1.92)	
**Treatment protocol**											
ST	404	87	0.83 (0.71–0.95)		<0.001					1	<0.001
CT	(82.3)	(17.7)	5.66 (1.94–9.38)							0.28 (0.20–0.37)	
**Period advanced diagnosis to register**											
Mean (SD)	6.12 (2.55)	5.46 (2.94)			<0.001						
< 3 months	60 (85.14)	40 (45.98)	0.93 (0.67–2.00)	3.17 (2.17–9.00)		115	115	0.54	0.14	1	0.844
3–6 months	204 (49.50)	22 (25.28)	0.83 (0.67–0.97)	8.17 (2.87–15.00)		406	406	0.46	0.08	1.31 (1.01–1.71)	
6–9 months	94 (27.23)	8 (9.20)	1.07 (0.67–1.67)	5.00 (0.73–8.00)		210	210	0.41	0.09	1.21 (0.89–1.65)	
>9 months	46 (39.11)	17 (19.54)	0.67 (0.44–1.77)	5.17 (200–9.00)		59	59	0.72	0.09	1.16 (0.82–1.63)	
**Status**											
Alive	22 (5.45%)	35 (40.23%)	0.000								
Dead	382 (94.55%)	52 (59.77%)	0.000								

All baseline information was obtained from **secondary data extracted from the hospital's electronic medical record systems.** Data on cancer stage, ECOG performance status, and baseline pain levels were not available.

There were 491 patients (296 males and 195 females) with CCA and HCC; there were 404 in the ST group (242 males and 162 females) and 87 in the CT group (54 males and 33 females). The mean ages of the ST group were 66.60, and the CT group was 65.64 years. Most patients (43.38%) were 70 years of age. More than 71.53% in the ST group received cancer chemotherapy and combinations, and 49.42% of the CT group also received palliative care. The mean point of diagnosis with advanced CCA, HCC to registration was 8.65 months for ST, and 5.32 months for CT. Most patients (38.49%) were registered at the palliative and/or cannabis care clinic, and 94.60% (ST), 59.80% (CT) had died by the end of the study. The total follow-up time for ST patients was 790 person-months, with a mortality rate of 48.35/100 person-years. For the CT group, follow-up was 476 person-months, a mortality rate of 10.9./100 person-years for CT (  Table 2).

**Table 2.** Baseline characteristics and survival outcomes of advanced cholangiocarcinoma patients by treatment group (Standard treatment [ST] vs. Cannabis treatment [CT]) (n=491).

Variable	Patient treatment group		Median survival time (months, 95% CI)		Person-time (months)	IR/100 person-month	HR_adj (95% CI)	P-value
	ST (n=404, %)	CT (n=87, %)	ST (n=404, %)	CT (n=87, %)				
Overall Survival			0.83 (0.71–0.95)	5.66 (1.94–9.38)			0.28 (0.20–0.37)	<0.001
Age <60	105 (26.0)	24 (27.6)	0.83 (0.60–1.00)	5.67 (2.87–15.00)	170/147	0.59/0.08	1.00	0.212
Age 60–69	121 (30.0)	28 (32.2)	0.93 (0.73–1.04)	3.27 (2.00–12.00	244/128	0.47/0.13	0.85 (0.66–1.09)	
Age ≥70	178 (44.1)	35 (40.2)	0.83 (0.67–1.27)	6.00 (2.33–10.03)	375/200	0.44/0.11	0.87 (0.68–1.09)	
Sex: Male	242 (60.0)	54 (62.1)	0.73 (0.67–0.93)	6.00 (3.07–10.03)	427/300	0.53/0.10	1.00	0.236
Sex: Female	162 (40.0)	33 (37.9)	0.97 (0.83–1.20)	3.50 (1.77–9.50)	362/175	0.42/0.11	0.89 (0.73–1.08)	
Cancer Treatment: Surgery	28 (6.9)	4 (4.6)	1.33 (0.30–2.50)	2.00 (1.83–10.00)	106/14	0.20/0.21	1.00	0.106
Chemotherapy	140 (34.7)	18 (20.7)	0.93 (0.73–1.00)	9.50 (5.17–15.00)	209/139	0.65/0.06	1.43 (0.93–2.20)	
Combined	149 (36.9)	22 (25.3)	0.83 (0.67–1.27)	7.00 (1.67–15.00)	311/121	0.45/0.09	1.27 (0.82–1.93)	
Palliative Care	87 (21.5)	43 (49.4)	0.73 (0.50–0.93)	3.07 (2.17–8.33)	162/201	0.51/0.14	1.23 (0.79–1.92)	
Time from Diagnosis to Registration <3 months	60 (14.9)	40 (46.0)	0.93 (0.67–2.00)	3.17 (2.17–9.00)	115/115	0.54/0.14	1.00	0.844
3–6 months	204 (49.5)	22 (25.3)	0.83 (0.67–0.97)	8.17 (2.87–5.00)	406/406	0.46/0.08	1.31 (1.01–1.71)	
6–9 months	94 (23.3)	8 (9.2)	1.07 (0.67–1.67)	5.00 (0.73–8.00)	210/210	0.41/0.09	1.21 (0.89–1.65)	
>9 months	46 (11.4)	17 (19.5)	0.67 (0.44–1.77)	5.17 (2.00–9.00)	59/59	0.72/0.09	1.16 (0.82–1.63)	
Status at Study End: Dead	382 (94.6)	52 (59.8)						

Abbreviations: ST, Standard treatment; CT, Cannabis treatment; HR, Hazard ratio; CI, Confidence interval; IR, Incidence rate. *Notes:*
• All data were obtained from secondary sources (hospital electronic medical records. • Median survival time is presented in months with 95% confidence intervals. Incidence rate is expressed per 100 person-months. Censored cases include patients alive at the study endpoint or lost to follow-up. Hazard ratios are adjusted for age, sex, type of cancer treatment, and period from diagnosis to registration using Cox proportional hazards regression. • HR<1 indicates a reduced hazard of death, and specifically interpret the HR for medical cannabis as indicating prolonged survival.

### Survival outcomes

**Figure 1.**
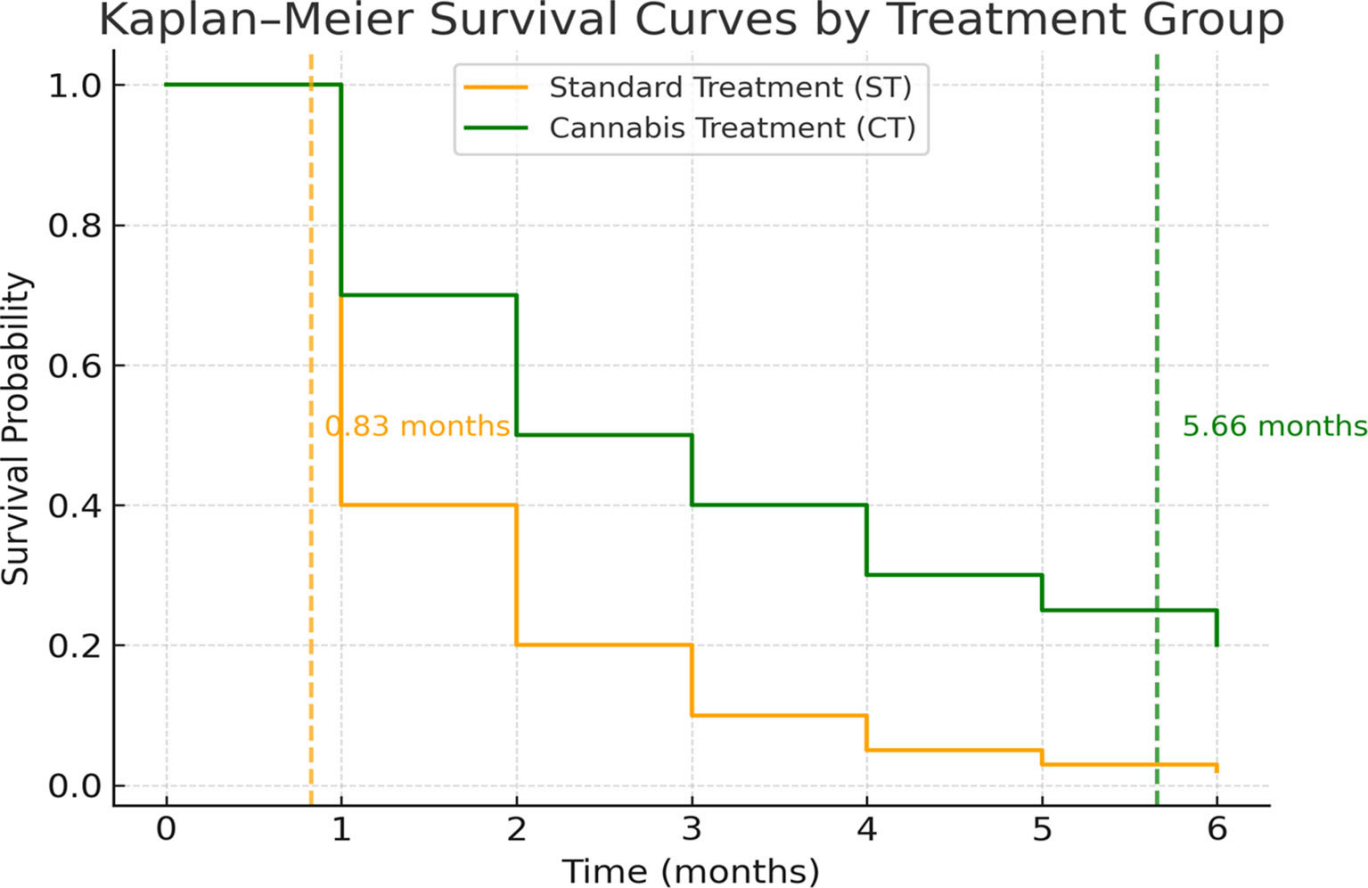
Kaplan–Meier survival curves for advanced cholangiocarcinoma (CCA) and hepatocellular carcinoma (HCC) patients receiving standard palliative care treatment (ST) and medicinal cannabis treatment (CT).

Survival outcomes are presented in  Table 2 and illustrated in  Figure 1. For ST patients, the total follow-up time was 790 person-months, with a mortality rate of 48.35 per 100 person-months. For CT patients, the total follow-up time was 476 person-months, with a mortality rate of 10.9 per 100 person-months.

**Table 3.** Multivariable Cox proportional hazards regression analysis for overall survival among patients with advanced cholangiocarcinoma (n = 491).

Variable	Adjusted HR	95% CI	p-value
Treatment group			
Standard treatment (ST)	Reference	—	—
Cannabis treatment (CT)	0.28	0.20–0.37	<0.001
Age group			
<60 years	Reference	—	—
60–69 years	0.85	0.66–1.09	0.212
≥70 years	0.87	0.68–1.09	0.236
Sex			
Male	Reference	—	—
Female	0.89	0.73–1.08	0.236
Type of prior cancer treatment			
Surgery	1.00	—	0.106
Chemotherapy	1.43	0.93–2.20	0.093
Combined treatment	1.27	0.82–1.93	0.272
Palliative care only	1.23	0.79–1.92	0.355
Time from diagnosis to registration			
<3 months	Reference	—	—
3–6 months	1.31	1.01–1.71	0.044
6–9 months	1.21	0.89–1.65	0.222
>9 months	1.16	0.82–1.63	0.398

Notes: All data were obtained from secondary sources (hospital electronic medical records). Adjusted hazard ratios were estimated using a Cox proportional hazards regression model including treatment group, age group, sex, type of prior cancer treatment, and time from diagnosis to registration as covariates. Data on cancer stage, ECOG performance status, pain scores, and quality of life were not available in the secondary dataset and were therefore not included in the model.

The median survival time was calculated from the **date of registration** at the palliative care or cannabis clinic to the date of death or the study endpoint (30 June 2021). The median survival time was 0.83 months (95% CI: 0.71–0.95) for the ST group and 5.66 months (95% CI: 1.94–9.38) for the CT group. Kaplan–Meier survival curves (  Figure 1) demonstrated significantly longer survival for the CT group compared with the ST group (log-rank test, p < 0.001).

### Multivariable analysis

In the Cox proportional hazards regression model adjusted for age, sex, and time from diagnosis to registration, receiving medicinal cannabis treatment was significantly associated with prolonged survival compared to standard treatment (adjusted HR = 0.28; 95% CI: 0.20–0.37; p < 0.001). Age, sex, and most types of prior cancer treatment were not significantly associated with overall survival. However, a time from diagnosis to registration of 3–6 months was associated with a higher risk of death compared to less than 3 months (adjusted HR = 1.31; 95% CI: 1.01–1.71; p = 0.044) (  Table 3).

## Discussion and Conclusion

In the present study, we examined the survival outcomes of patients with advanced cholangiocarcinoma (CCA) or hepatocellular carcinoma (HCC) receiving either standard palliative care treatment (ST) or medicinal cannabis treatment (CT) across tertiary and secondary hospitals in five provinces. Survival time was calculated from the date of registration at the palliative or cannabis clinic until death or censoring. The CT group demonstrated a markedly longer median survival time (5.66 months) compared with the ST group (0.83 months).

These findings are generally consistent with previous research in Northeastern Thailand, where patients receiving only supportive treatment had a median survival of 4.3 months after diagnosis. ^18^ Moreover, patients diagnosed at an advanced stage were almost twice as likely to die (HR: 1.8; 95% CI: 1.1–2.9). ^23– 25^ However, our results contrast with Bar-Sela et al., ^14^ who reported shorter overall survival among advanced cancer patients using cannabis compared with non-users. This discrepancy may reflect differences in baseline characteristics, timing of cannabis initiation, disease stage, and access to other systemic treatments.

In the adjusted Cox regression model (  Table 2), receiving medicinal cannabis treatment was significantly associated with prolonged survival (adjusted HR = 0.28; 95% CI: 0.20–0.37; p < 0.001). However, the set of adjustment variables was limited to age, sex, type of cancer treatment, and time from diagnosis to registration due to the constraints of using secondary data. ^26^ Important clinical parameters such as performance status, tumor burden, comorbidities, and detailed treatment history could not be included, and residual confounding is therefore possible. ^27^

Baseline differences in care pathways may partly explain the observed survival advantage in the CT group. Many ST patients were referred to palliative clinics late in their disease trajectory, often after exhausting surgery, chemotherapy, or combination regimens. ^28^ In contrast, CT patients—often older and treatment-naïve—were frequently registered directly at cannabis clinics in community hospitals following imaging or biopsy confirmation of advanced metastases. ^29^ Some of these patients also received chemotherapy concurrently with cannabis, which may have contributed to extended survival. ^30^

From a biological perspective, cannabinoids may improve survival indirectly by alleviating symptoms (e.g., pain, anorexia, nausea), enhancing nutritional intake, improving sleep, and enabling greater tolerance to systemic therapy. ^11, 13, 31^ Preclinical studies also suggest potential anti-tumor effects via apoptosis induction, inhibition of angiogenesis, and suppression of tumor proliferation, ^28^ although these effects remain to be validated in large-scale clinical trials. Taken together, the observed survival benefit in the CT group may be partly attributable to both patient- and treatment-related covariates, as well as the potential symptom-modulating and anti-tumor mechanisms of cannabinoids demonstrated in preclinical studies. ^11, 13, 15, 18^ This integrated interpretation underscores the multifactorial nature of survival outcomes in advanced CCA/HCC.

To our knowledge, this is the first multicenter Thai study to compare survival outcomes between ST and CT in advanced CCA/HCC patients treated with standardized, FDA-approved medicinal cannabis products under physician supervision. ^17– 33^ Our findings support the integration of medicinal cannabis into palliative care, ^33^ especially in community settings with limited oncology resources.

Strengths of this study include the relatively large sample size, multi-level hospital participation, and the use of survival analysis adjusted for available covariates. Limitations include the retrospective design, reliance on secondary data, ^25^ incomplete information on cannabis dosage/formulation/adherence, and the inability to adjust for important prognostic factors. Consequently, while the association between cannabis treatment and improved survival is compelling, causality cannot be established without prospective randomized controlled trials. ^26^

## Author contributions

N.P. contributed to the research design, data collection, and manuscript writing. P.P., A.W., contributed to data collection and revised the manuscript, and R. W contributed to research administrator, research design, data collection, review and revised the manuscript.

## Statements

### Statement of ethics

This study adhered to the principles of the Declaration of Helsinki and the International Council for Harmonisation (ICH) Good Clinical Practice Guidelines. It was a retrospective cohort study using secondary data from hospital medical record systems and reporting databases from oncology and cannabis clinics. All ethics committees waived the requirement for individual informed consent, as all patient data were anonymized before analysis. No direct patient contact, treatment modification, or additional intervention was performed. No animal experiments were conducted.

### Consent to participate statement

Given the retrospective nature of the study, the requirement for written informed consent was waived by the Maha Sarakham University Human Research Ethics Committee, the Roi Et Regional Hospital Ethics Committee, and the Buriram Regional Hospital Ethics Committee. All decisions regarding consent waivers were made in accordance with applicable regulations and ethical guidelines.

### Consent for publication

Not applicable, as no identifiable individual data are included in this publication.

### Compliance with guidelines

All methods were carried out according to relevant guidelines and regulations.

## Data Availability

Patient data were available in the medical records room of the Roi-Et Regional Hospital, Burirum Regional Hospital, Surin Provincial Hospital, Sawang Dandin Crown Prince Hospital, Panna Nikhom Hospital, and Pana Hospital. The datasets generated and/or analysed during the current study are not publicly available because they are files in the medical records room in our hospital, but they are available from the corresponding author upon reasonable request. Figshare: Data_survival_cannabis. https://doi.org/10.6084/m9.figshare.20101193.v1. ^34^ Figshare: F1000_survival_table1_narisara_ranee. https://doi.org/10.6084/m9.figshare.20486913.v1. ^35^ Data are available under the terms of the Creative Commons Attribution 4.0 International license (CC-BY 4.0).

## References

[1] Azizi AA Hadjinicolaou AV Goncalves C : Update on the Genetics of and Systemic Therapy Options for Combined Hepatocellular Cholangio carcinoma. *Front Oncolog.* 2020;10: 570958. 10.3389/fonc.2020.570958 33102226 PMC7545907

[2] Wang J Li E Yang H : Combined hepatocellular cholangiocarcinoma: a population level analysis of incidence and mortality trends. *World J Surg Onc.* 2019 Dec;17(1):43. 10.1186/s12957-019-1586-8 30813932 PMC6394104

[3] Kamsa-Ard S Luvira V Suwanrungruang K : Cholangiocarcinoma incidence and survival in Khon Kaen, Thailand: A population-based study. *BMC Cancer.* 2019;19:291. 30078813 10.2188/jea.JE20180007 PMC6445798

[4] Banales JM Cardinale V Carpino G : Expert consensus document: Cholangiocarcinoma: current knowledge and future perspectives consensus statement from the European Network for the Study of Cholangiocarcinoma (ENS-CCA). *Nat Rev Gastroenterol Hepatol.* 2020;17(9):557–588. 10.1038/s41575-020-0310-z 27095655

[5] Valle JW Kelley RK Nervi B : Biliary tract cancer. *Lancet.* 2016;388(10061):1469–1484.

[6] Bray F Ferlay J Soerjomataram I : Global cancer statistics 2018: GLOBOCAN estimates of incidence and mortality worldwide for 36 cancers in 185 countries. *CA Cancer J Clin.* 2018 Nov;68(6):394–424. doi: 10.3322/caac.21492. Epub 2018 Sep 12. Erratum in: CA Cancer J Clin. 2020 Jul;70(4):313. 10.3322/caac.21609 30207593

[7] Kawasaki S Imamura H Kobayashi A : Results of 100 liver resections for combined hepatocellular and cholangiocarcinoma. *J Hepatobiliary Pancreat Surg.* 2015;10(1):31–39.

[8] Kim KH Lee SG Park EH : Surgical treatments and prognoses of combined hepatocellular and cholangiocarcinoma of the liver. *J Gastrointest Surg.* 2012;16(3):487–496. 10.1245/s10434-008-0278-3 19130133

[9] Luvira V : Outcomes of surgical resection for cholangiocarcinoma in Northeastern Thailand. *Asian Pac J Cancer Prev.* 2017;18(2):565–572.

[10] Rizvi S Khan SA Hallemeier CL : Cholangiocarcinoma — evolving concepts and therapeutic strategies. *Nat Rev Clin Oncol.* 2018;15(2):95–111. 10.1038/nrclinonc.2017.157 28994423 PMC5819599

[11] Whiting PF Wolff RF Deshpande S : Cannabinoids for medical use: A systematic review and meta-analysis. *JAMA.* 2015;313(24):2456–2473. 10.1001/jama.2015.6358 26103030

[12] Johnson JR Burnell-Nugent M Lossignol D : Multicenter, double-blind, randomized, placebo-controlled, parallel-group study of the efficacy, safety, and tolerability of THC: CBD extract and THC extract in patients with intractable cancer-related pain. *J Pain Symptom Manage.* 2013;46(2):207–218. 10.1016/j.jpainsymman.2012.07.014 19896326

[13] Phansila N Sittiwet C Wongkongdech R : Comparison of effects of medicinal cannabis or standard palliative care on quality of life of patients with cholangiocarcinoma in Northeast Thailand [version 2; peer review: 1 approved, 1 approved with reservations]. *F1000Research.* 2025;11:20. 10.12688/f1000research.75060.2

[14] Bar-Sela G Vorobeichik M Drawsheh S : The medical necessity for medicinal cannabis in cancer patients: Lessons from clinical experience. *Support Care Cancer.* 2013;21(7):1905–1910.

[15] Ministry of Public Health, Thailand : *Cannabis-based medicine in palliative care: National policy and guidelines.* Nonthaburi: MOPH;2019.

[16] Chokevivat V Pongpirul K Buranapraditkun S : Medical cannabis in Thailand: Legal framework and policy implementation. *J Med Assoc Thai.* 2021;104(7):1065–1072.

[17] World Law Group .2020Global Cannabis Guide – Thailand [Internet].2020Oct 16 [cited 2021 Apr 18]. Reference Source

[18] Khuntikeo N Loilome W Thinkhamrop B : A comprehensive public health conceptual framework and strategy to effectively combat cholangiocarcinoma in Thailand. *PLoS Negl Trop Dis.* 2016;10(1):e0004293. 10.1371/journal.pntd.0004293 26797527 PMC4721916

[19] Dasu N Soloway M Thuluvath AJ : Patients with cannabis use and cholangiocarcinoma have lower in-hospital mortality: analysis of the National Inpatient Sample. *Am J Gastroenterol.* 2022;117(Suppl):S99. 10.14309/01.ajg.0000864854.61961.5a

[20] Jeong S : Cannabidiol and cannabigerol inhibit cholangiocarcinoma cell growth via apoptosis, autophagy, and cell cycle arrest. *Front Pharmacol.* 2022;13:924221. 10.3389/fphar.2022.924221

[21] Notarnicola M : Inhibitory effects of cannabinoids on cholangiocarcinoma cell proliferation and migration. *Cancer Invest.* 2009;27(6):549–555. 10.1080/07357900802543359 19229700

[22] Khan SA : Guidelines for the diagnosis and treatment of cholangiocarcinoma: an update. *Gut.* 2022;71(1):1–29. 10.1136/gutjnl-2021-325087 22895392

[23] Sripa B Pairojkul C Thinkhamrop B : The tumor registry of cholangiocarcinoma in Northeastern Thailand: A report of 198 cases. *Asian Pac J Cancer Prev.* 2020;21(3):703–709.

[24] Thunyaharn N Promthet S Wiangnon S : Survival of Cholangiocarcinoma Patients in Northeastern Thailand after Supportive Treatment. *Asian Pac. J. Cancer Prev.* 2013 Nov 30;14:7029–7032. 10.7314/APJCP.2012.14.11.7029 24377644

[25] Woradet S Songserm N Promthet S : Health-Related Quality of Life and Survival of Cholangiocarcinoma Patients in Northeastern Region of Thailand. *PLoS One.* 2016;11(9):e0163448. 10.1371/journal.pone.0163448 27685448 PMC5042427

[26] World Health Organization : *Planning and implementing palliative care services: A guide for programme managers.* Geneva: WHO;2016.

[27] Hernán MA Robins JM : *Causal Inference: What If.* Boca Raton: Chapman & Hall/CRC;2020.

[28] Palliative Care Society of Thailand : *National palliative care guidelines.* Bangkok: Palliative Care Society of Thailand;2021.

[29] National Cancer Institute Thailand : *Cancer registry annual report.* Bangkok: NCI Thailand;2022.

[30] Schroeder A McPartland JM : *Cannabis science and policy: Examining the science behind the policy.* New York: Oxford University Press;2018.

[31] Maida V Daeninck PJ : A user’s guide to cannabinoid therapies in oncology. *Curr Oncol.* 2016;23(6):398–406. 10.3747/co.23.3487 28050136 PMC5176373

[32] Velasco G Sánchez C Guzmán M : Anticancer mechanisms of cannabinoids. *Curr Oncol.* 2016;23(S2):S23–S32. 10.3747/co.23.3080 PMC4791144 27022311

[33] Thai Food and Drug Administration: *Guidelines for medicinal cannabis use in palliative care.* Nonthaburi: Thai FDA;2023.

[34] Phansila N Pansila P Wongkongdech A : Data_survival_cannabis. figshare. [Dataset].2022. 10.6084/m9.figshare.20101193.v1

[35] Phansila N : F1000_survival_table1_narisara_ranee. figshare. [Dataset].2022. 10.6084/m9.figshare.20486913.v1

