# Supplementary material for: Survival rate of patients with combined hepatocellular cholangiocarcinoma receiving medical cannabis treatment: A retrospective, cohort comparative study
Source: F1000Res. 2025 Oct 3;11:1212. Originally published 2022 Oct 25. [Version 3] doi: 10.12688/f1000research.123250.3 (PMC12519070; doi:10.12688/f1000research.123250.3)
Supplement: Peer review history

# Reviewer report: Reviewer response for version 3

**DOI:** 10.5256/f1000research.186703.r420117

**Published:** 2025-10-14

**Authors:** Nuttapong Ngamphaiboon

**Affiliations:** 1 Mahidol University, Bangkok, Thailand

Many major concerns are not addressed properly, led to difficulty to make conclusions from the data due to multiple confounders.

Is the work clearly and accurately presented and does it cite the current literature?

No

If applicable, is the statistical analysis and its interpretation appropriate?

Partly

Are all the source data underlying the results available to ensure full reproducibility?

No

Is the study design appropriate and is the work technically sound?

No

Are the conclusions drawn adequately supported by the results?

No

Are sufficient details of methods and analysis provided to allow replication by others?

No

Reviewer Expertise:

Oncology

I confirm that I have read this submission and believe that I have an appropriate level of expertise to state that I do not consider it to be of an acceptable scientific standard, for reasons outlined above.

# Reviewer report: Reviewer response for version 3

**DOI:** 10.5256/f1000research.186703.r420115

**Published:** 2025-10-13

**Authors:** Nat Na-Ek

**Affiliations:** 1 Division of Social and Administration Pharmacy, Department of Pharmaceutical Care, School of Pharmaceutical Sciences, University of Phayao, Mueang Phayao District, Phayao, Thailand

This manuscript addresses an important clinical question, but several major methodological flaws remain unresolved as outlined below:

 It is unclear whether the study population consists of cholangiocarcinoma (CCA), combined hepatocellular–cholangiocarcinoma (cHCC-CCA), or either CCA or HCC. In the abstract, CCA is mentioned, whereas in the introduction cHCC-CCA is discussed. Later, in the methods section, patients with advanced CCA or HCC are included. This inconsistency confuses readers and substantially undermines the trustworthiness of the work.

 Although my E-value analysis suggests that unmeasured confounding is unlikely to fully explain away the observed association (adjusted HR = 0.28, 95% CI = 0.20, 0.37; E-value 6.602 for the point estimate and 4.848 for the confidence interval), the magnitude of the effect warrants careful scrutiny. In clinical epidemiology, it is rare for a single unmeasured factor to be associated with both exposure and outcome by risk ratios exceeding five- to seven-fold each, especially after adjustment for age and sex. This indicates that residual confounding alone is an improbable explanation for the observed results. However, several other alternative explanations could plausibly account for such a pronounced effect size. Firstly, survival (immortal) time bias may have arisen because the cannabis group was registered earlier, whereas controls experienced longer lag periods before registration, effectively guaranteeing a survival advantage for cannabis users. This may not be adequately controlled by simply adjusting for the lag period as a categorical variable. Secondly, selective inclusion is a concern: if healthier or more engaged patients were more likely to receive or report cannabis use, the apparent benefit could reflect reverse causation rather than treatment efficacy. Thirdly, the removal of the patient flow diagram and the unclear handling of missing data reduce transparency, raising the possibility that exclusions disproportionately affected sicker controls. Collectively, these biases offer more credible explanations for the striking hazard ratio reported than unmeasured confounding alone. The authors should therefore conduct sensitivity analyses, such as landmark analyses, time-dependent modelling, and transparent reporting of exclusions and missingness, before drawing strong conclusions about treatment effects. In addition, please verify the correctness of the percentages reported in Table 1.

 The rationale for adopting a two-step approach is unclear. Univariable selection should be avoided (TRIPOD) as it can lead to omission of important covariates and model misspecification. Best practice in covariate selection involves the use of DAGs or prior knowledge, rather than relying solely on statistical significance.

 In Figure 1, the dashed vertical lines do not appear to represent the median survival time of each group. By definition, the median survival should correspond to the time point at which 50% of the population remains alive. Please check the correctness of the Kaplan–Meier curve.

 In the abstract:

 Please include a specific objective or research gap in the final sentence of the background subsection.

 Please explicitly state the adjustment factors in the methods subsection.

 In the results subsection, follow-up time should generally be reported as the median with corresponding interquartile range rather than as incidence density (person-time). Please revise accordingly.

 In the conclusion subsection, please avoid implying causality. For example, the sentence “Medical cannabis increased overall survival rates among CCA patients” should be rephrased as “Medical cannabis was associated with increased overall survival rates among CCA patients”

Is the work clearly and accurately presented and does it cite the current literature?

Partly

If applicable, is the statistical analysis and its interpretation appropriate?

Partly

Are all the source data underlying the results available to ensure full reproducibility?

Partly

Is the study design appropriate and is the work technically sound?

Yes

Are the conclusions drawn adequately supported by the results?

Partly

Are sufficient details of methods and analysis provided to allow replication by others?

Partly

Reviewer Expertise:

NA

I confirm that I have read this submission and believe that I have an appropriate level of expertise to confirm that it is of an acceptable scientific standard, however I have significant reservations, as outlined above.

# Reviewer report: Reviewer response for version 2

**DOI:** 10.5256/f1000research.180104.r377014

**Published:** 2025-09-10

**Authors:** Selamat Budijitno

**Affiliations:** 1 Department of Surgery, Dr. Kariadi Hospital, Faculty of Medicine, Universitas Diponegoro, Semarang, Central Java, Indonesia

I approve for this "version 2" of the article.

Is the work clearly and accurately presented and does it cite the current literature?

Yes

If applicable, is the statistical analysis and its interpretation appropriate?

Yes

Are all the source data underlying the results available to ensure full reproducibility?

Partly

Is the study design appropriate and is the work technically sound?

Yes

Are the conclusions drawn adequately supported by the results?

Yes

Are sufficient details of methods and analysis provided to allow replication by others?

Partly

Reviewer Expertise:

Surgical Oncology, biomolecular, Immunology, epidemiology

I confirm that I have read this submission and believe that I have an appropriate level of expertise to confirm that it is of an acceptable scientific standard.

# Reviewer report: Reviewer response for version 2

**DOI:** 10.5256/f1000research.180104.r400722

**Published:** 2025-08-12

**Authors:** Dima Malkawi

**Affiliations:** 1 University of Colorado, Denver, Colorado, USA

**Introduction**:

 - The second paragraph of the introduction focuses only on CC but this study focuses on cHCC-CC so the introduction needs to be expanded to include both. 

 - The third paragraph mentions a standard and a new treatment; it would be good to expand on both here to know what the difference is between the two and why that may be relevant 

 - Would use the intro to discuss the logic behind this study and why it is impactful or necessary. Are there big differences between the two methods? 

**Methods:**

 - The flow diagram is not adding much to this manuscript

 - Would recommend CONSORT diagram instead 

 - No information is provided on treatment protocols including dosing, frequency etc

 - Patients in CT vs. ST may differ in unmeasured ways (e.g., baseline performance status, comorbidities, tumor burden, liver function). For example, the CT group had much lower mortality rates and higher follow-up times, but it’s unclear whether this is due to the treatment or differences in baseline prognosis.

 - There is no mention of propensity score matching or multivariable adjustment for critical prognostic variables beyond age, sex, and treatment.

 Without controlling for these confounders, the observed survival difference may be overestimated

 - Consider adjusting for additional baseline variables in the Cox model (e.g., ECOG performance status, Child-Pugh score, metastasis status, comorbidities, prior treatments). If unavailable, acknowledge this as a serious limitation

 - Your inclusion criteria state “newly diagnosed with CCA or HCC,” yet the title and aim focus on *combined hepatocellular cholangiocarcinoma (cHCC-CC)*

**- **unclear whether all patients indeed had **cHCC-CC**, or whether you included *either* HCC or CCA

**- **Clarify whether patients were pathologically confirmed as cHCC-CC or diagnosed clinically as HCC/CCA. If both were included, justify combining them.

 - The survival times reported (ST: 0.83 months) seem *extremely short*, raising the possibility that these were highly selected end-stage patients.

**Results**: 

 - The authors state that they tested the proportional hazards assumption but do not report the findings. They also state they did kaplan meier survival curves which are also not reported

 - The data is reported as a causal relationship which is extremely misleading 

 - No data presented on differences in treatment populations 

 - Chemotherapy is initially defined as CT but then later Cannabis Therapy is also defined as CT causing some confusion 

 - Data on treatment types and staging is not discussed and thus comparisons between the groups may not be accurate or reflective of true outcomes, and possibly considered invalid

 Overall:

 - The extremely high CC incidence in your region is discussed, but the CT effect may not translate to other settings.

 - Cannabis preparations (THC:CBD ratios, dosing) are specific to Thai FDA-approved products — this needs more detail for reproducibility.

Is the work clearly and accurately presented and does it cite the current literature?

Partly

If applicable, is the statistical analysis and its interpretation appropriate?

No

Are all the source data underlying the results available to ensure full reproducibility?

No

Is the study design appropriate and is the work technically sound?

No

Are the conclusions drawn adequately supported by the results?

No

Are sufficient details of methods and analysis provided to allow replication by others?

No

Reviewer Expertise:

surgical oncology, outcomes

I confirm that I have read this submission and believe that I have an appropriate level of expertise to state that I do not consider it to be of an acceptable scientific standard, for reasons outlined above.

# Reviewer report: Reviewer response for version 2

**DOI:** 10.5256/f1000research.180104.r396266

**Published:** 2025-08-11

**Authors:** Nuttapong Ngamphaiboon

**Affiliations:** 1 Mahidol University, Bangkok, Thailand

The study contains major flaws in both design and methodology. The conclusions presented in the current version of the manuscript may be significantly biased due to the lack of detailed patient characteristics and treatment-related information.

**Major Comments:**

1. Given that cannabinoids are primarily used in palliative care to alleviate pain, reduce nausea, and stimulate appetite, the manuscript should clearly state the primary outcomes of palliative use such as symptom improvement of cannabinoid use in this study.
2. Detailed information regarding cannabinoid use should be provided, including dosage, duration, mode of administration, and patient compliance.
3. If survival outcomes are to be reported, critical clinical data must be included—such as ECOG performance status, cancer staging at the time of treatment, chemotherapy regimens, number of cycles, dosing details, relative dose intensity, and radiation therapy use. Without these essential details, the conclusion that "medical cannabis increased overall survival rates among CCA patients" cannot be considered reliable.
4. Kaplan–Meier curves for overall survival (OS) and progression-free survival (PFS) should be presented to support the survival analysis.

Is the work clearly and accurately presented and does it cite the current literature?

No

If applicable, is the statistical analysis and its interpretation appropriate?

Partly

Are all the source data underlying the results available to ensure full reproducibility?

No

Is the study design appropriate and is the work technically sound?

No

Are the conclusions drawn adequately supported by the results?

No

Are sufficient details of methods and analysis provided to allow replication by others?

No

Reviewer Expertise:

Oncology

I confirm that I have read this submission and believe that I have an appropriate level of expertise to state that I do not consider it to be of an acceptable scientific standard, for reasons outlined above.

# Response

**Published:** 2025-08-15

**Authors:** Narisara Phansila

**Affiliations:** Mahasarakham University, Thailand

**Reviewer’s Comment – Overall assessment of major flaws in design and methodology**

*The study contains major flaws in both design and methodology. The conclusions presented in the current version of the manuscript may be significantly biased due to the lack of detailed patient characteristics and treatment-related information.*

**Response:** We acknowledge the inherent limitations of our retrospective design and the use of secondary data. Our dataset was obtained from hospital records and contained survival status, selected patient characteristics, and treatment timing variables (e.g., date of diagnosis, date of registration for treatment). Detailed clinical information such as ECOG performance status, cancer staging, chemotherapy regimen details, dosing, and radiation therapy use was not captured in the database. We have explicitly stated these limitations in the revised manuscript, reframed our conclusions to avoid causal inference, and presented our findings as preliminary evidence requiring further validation through prospective studies.

**Reviewer’s Comment 1:**

*Given that cannabinoids are primarily used in palliative care to alleviate pain, reduce nausea, and stimulate appetite, the manuscript should clearly state the primary outcomes of palliative use such as symptom improvement of cannabinoid use in this study.*

**Response:** We agree that palliative outcomes such as symptom improvement are important; however, these were not available in the secondary data used for this analysis. We have clearly acknowledged this limitation in the revised manuscript and clarified that our study focuses exclusively on survival outcomes derived from available records.

**Reviewer’s Comment 2:**

*Detailed information regarding cannabinoid use should be provided, including dosage, duration, mode of administration, and patient compliance.*

**Response:** We have added all available treatment details to the Methods section, including the product type (Thai FDA-approved oral cannabis oil extract), THC:CBD ratios, and initial prescribed dosage. Information on treatment duration, dose adjustments, and patient compliance was not recorded in the database and is acknowledged as a limitation.

**Reviewer’s Comment 3:**

*If survival outcomes are to be reported, critical clinical data must be included—such as ECOG performance status, cancer staging at the time of treatment, chemotherapy regimens, number of cycles, dosing details, relative dose intensity, and radiation therapy use. Without these essential details, the conclusion that "medical cannabis increased overall survival rates among CCA patients" cannot be considered reliable.*

**Response:** We agree that these clinical variables are important prognostic factors; however, they were not captured in the secondary database used for this study. Available data were limited to demographic characteristics, timing variables (diagnosis date, registration date), treatment type (cannabis vs. standard care), and survival status. We have emphasized these limitations in the revised manuscript and reframed our conclusions to state that medicinal cannabis treatment was associated with prolonged survival, while noting that causality cannot be inferred.

**Reviewer’s Comment 4:**

*Kaplan–Meier curves for overall survival (OS) and progression-free survival (PFS) should be presented to support the survival analysis.*

**Response:** We have added Kaplan–Meier curves for OS (Figure 1). PFS could not be analyzed because progression dates were not consistently recorded in the dataset. This limitation is stated in both the Methods and Limitations sections.

**Competing Interests:**

 We confirm that the authors have no competing interests to declare.

# Reviewer report: Reviewer response for version 2

**DOI:** 10.5256/f1000research.180104.r383930

**Published:** 2025-06-03

**Authors:** Ueamporn Summart

**Affiliations:** 1 Faculty of Nursing, Roi Et Rajabhat University, Tha Muang, Thailand

Title: Survival rate of patients with combined hepatocellular cholangiocarcinoma receiving medical cannabis treatment: A retrospective, cohort comparative study

Upon completing a comprehensive analysis of all your amended manuscripts, I would like to express my gratitude for your diligent efforts in enhancing and elucidating your manuscripts. In my opinion, there are several areas where the authors may improve in order to enhance the reader's understanding of their work, as outlined below:

 Abstract:

 Conclusions: “The medical cannabis increased overall survival rates among CCA patients.”

 In my opinion, we do not conclude that only the effect of medical cannabis increased overall survival rates among this population because this study design is not supported. Furthermore, the study did not control or include other confounding factors that could affect the survival outcome.

 Introduction:

 1) Suggest the authors clarify the population of this study. (recurving palliative or curative treatment) and also describe more information about these treatments that are specific for this population before mentioning the use of medical cannabis treatment in this study. (Some details are missing between paragraph 2 and paragraph 3, such as treatment for palliative CCA or specific survival rates and other treatment for this population in Thailand.

 2) Suggest the authors mentioned the rationale (literature reviewed) of the covariates and the medical cannabis treatment used in this study from previous studies.

 Statistical analysis:

 1) Due to the retrospective cohort design, this study aimed to compare the survival rate between 2 treatments, so it is better to compare baseline characteristics of the participants because baseline imbalance among this sample will lead to selection biases.

 2) To compare the survival rate between two groups using the log-rank test, the author should mention the assumption for this statistic before using it.3.3) Suggest the authors used a Kaplan-Meier graph to compare survival outcomes between two treatments following covariates in this study, such as age and cancer treatments. 3.4) For Cox proportional hazard statistics, suggest the authors mention the assumption of these statistics and steps of analysis such as univariable analysis and multivariable analysis. (I cannot find this step, but the authors mentioned these steps in the discussion part.).

 Results:

 1) I agree with reviewer 1 to separate the table into 3 tables as follows: Table 1 compares baseline characteristics. Normally, the authors presented the covariate in the table following the patient treatment group, so it is not the covariate. Suggest separating this table out of the tables that present the study objectives.

 2) Suggest the authors present survival outcomes in another table along with Kaplan-Meier graph comparing survival outcomes between two treatments following covariate.

 3) Suggest the authors present a table for the Cox proportional hazard ratio to present crude HR and adjusted HR, followed by steps of multivariable analysis. In addition, for the HR less than 1, please interpret this result for the readers.

 Discussions:

 Suggest the authors discuss following all of the covariates used in this study. In addition, the mechanism of medical cannabis treatment should be mentioned, whether it increased overall survival rates among CCA patients.

Is the work clearly and accurately presented and does it cite the current literature?

Partly

If applicable, is the statistical analysis and its interpretation appropriate?

Partly

Are all the source data underlying the results available to ensure full reproducibility?

Partly

Is the study design appropriate and is the work technically sound?

Yes

Are the conclusions drawn adequately supported by the results?

Partly

Are sufficient details of methods and analysis provided to allow replication by others?

Partly

Reviewer Expertise:

Nursing and Public Health

I confirm that I have read this submission and believe that I have an appropriate level of expertise to confirm that it is of an acceptable scientific standard, however I have significant reservations, as outlined above.

# Response

**Published:** 2025-08-15

**Authors:** Narisara Phansila

**Affiliations:** Mahasarakham University, Thailand

**Reviewer’s Comment – Abstract**

*Conclusions: “The medical cannabis increased overall survival rates among CCA patients.” In my opinion, we do not conclude that only the effect of medical cannabis increased overall survival rates among this population because this study design is not supported. Furthermore, the study did not control or include other confounding factors that could affect the survival outcome.*

**Response:**

 We appreciate this important observation. The Abstract conclusion has been revised to avoid implying causality. The new conclusion now reads:

 “In this retrospective cohort, medicinal cannabis use was associated with longer overall survival among advanced CCA patients; however, causality cannot be inferred due to the retrospective design and residual confounding.”

 This rewording aligns with the study design and acknowledges the potential impact of unmeasured confounding factors.

*(Page 1, Lines 36–39)*

**Reviewer’s Comment – Introduction (1)**

*Clarify the population of this study (receiving palliative or curative treatment) and describe more information about these treatments specific to this population before mentioning medical cannabis. Include survival rates and treatments for CCA in Thailand.*

**Response:**

 We have clarified that all patients in this study were diagnosed with advanced disease and were registered at palliative care or cannabis clinics. No patients received curative-intent treatment. We have expanded the description of standard palliative care and systemic treatment options available in Thailand, including expected survival times, supported by local and international references.

*(Page 2, Lines 70–76, 78–79)*

**Reviewer’s Comment – Introduction (2)**

*Mention the rationale (literature reviewed) of the covariates and medical cannabis treatment used in this study from previous studies.*

**Response:**

 We have added a paragraph explaining the rationale for each covariate (age, sex, type of cancer treatment, time from diagnosis to registration) based on prior survival studies. We also strengthened the rationale for examining medicinal cannabis by summarizing evidence from Thai and global literature on its palliative benefits and possible anti-cancer effects.

*(Page 2, Lines 70–79)*

**Reviewer’s Comment – Statistical Analysis (1)**

*Compare baseline characteristics to address possible selection bias.*

**Response:**

 We have compared baseline characteristics between treatment groups in the new Table 1 and discussed differences that may indicate potential selection bias.

*(Page 4, Table 1)*

**Reviewer’s Comment – Statistical Analysis (2)**

*Mention the assumption for the log-rank test.*

**Response:**

 We have explicitly stated the assumptions for the log-rank test (proportional hazards, non-informative censoring) in the Statistical Analysis section.

*(Methods: Statistical Analysis)*

**Reviewer’s Comment – Statistical Analysis (3)**

*Use Kaplan–Meier graph to compare survival outcomes between treatments by covariates (e.g., age, cancer treatments).*

**Response:**

 We have added stratified Kaplan–Meier survival curves by age group (<65 vs ≥65) and prior cancer treatment (yes/no) in the supplementary material. Figure 1 presents the overall survival curves for the two treatment groups.

*(Figure 1; Supplementary Figures)*

**Reviewer’s Comment – Statistical Analysis (4)**

*Mention the assumption of Cox regression and steps of analysis (univariable and multivariable).*

**Response:**

 We have described the proportional hazards assumption checks (Schoenfeld residuals, log-minus-log plots) and outlined the two-step analysis process: univariable screening (p<0.20) followed by multivariable Cox regression adjusting for selected covariates.

*(Methods: Statistical Analysis)*

**Reviewer’s Comment – Results (1)**

*Separate tables: Table 1 for baseline characteristics, separate from tables presenting study objectives.*

**Response:**

 We have reorganized the results into separate tables:

- **Table 1:** Baseline characteristics by treatment group
- **Table 2:** Survival outcomes (median survival, person-time, log-rank test)
- **Table 3:** Cox regression results (crude and adjusted HRs)
*(Pages 4–6, Tables 1–3)*

**Reviewer’s Comment – Results (2)**

*Present survival outcomes in another table along with Kaplan–Meier graph by covariates.*

**Response:**

 Survival outcomes are now presented in Table 2 and illustrated in Kaplan–Meier curves (Figure 1). Stratified curves by age and prior cancer treatment are included in the supplementary material.

**Reviewer’s Comment – Results (3)**

*Present Cox proportional hazard results in a table with crude and adjusted HR, interpret HR<1.*

**Response:**

 Table 3 presents both crude and adjusted HRs, with interpretation provided in the Results section. We explain that HR<1 indicates a reduced hazard of death, reflecting longer survival in that group.

*(Page 6, Table 3)*

**Reviewer’s Comment – Discussion**

*Discuss all covariates and the mechanism of medical cannabis treatment.*

**Response:**

 We have discussed each covariate’s association with survival outcomes, supported by relevant literature. Additionally, we expanded the discussion on potential mechanisms of medicinal cannabis, including symptom relief (pain, nausea, appetite, sleep) and preclinical evidence for anti-tumor activity (apoptosis induction, angiogenesis inhibition, tumor proliferation suppression).

*(Pages 6–7, Discussion)*

# Reviewer report: Reviewer response for version 2

**DOI:** 10.5256/f1000research.180104.r377013

**Published:** 2025-05-10

**Authors:** Nat Na-Ek

**Affiliations:** 1 Division of Social and Administration Pharmacy, Department of Pharmaceutical Care, School of Pharmaceutical Sciences, University of Phayao, Mueang Phayao District, Phayao, Thailand

Thank you for the revised version of the manuscript. However, it remains difficult to determine where amendments have been made in response to my previous comments. I would greatly appreciate it if the authors could provide a point-by-point response to each of my earlier comments, as a general summary is insufficient for a thorough review.

 Upon reviewing the revised manuscript, I noted several issues that appear to remain unaddressed, with no accompanying explanation. For example:

 - In the abstract, the authors continue to use the term “multivariate analysis” rather than the more appropriate “multivariable analysis”. While this is a relatively minor issue, it reflects a lack of attention to detail.

 - More importantly, in the final sentence of the results section and in the conclusion of the abstract, the authors continue to imply a causal relationship between medical cannabis and survival rates. For instance, statements such as “Therefore, CT had a reduced probability of dying from the disease” and “The medical cannabis increased overall survival rates among CCA patients” suggest causality. How can the authors be certain that the observed association is attributable solely to medical cannabis, rather than to other potential explanations?

 - Regarding the patient flow diagram, the figure presented appears to be a theoretical illustration more suited to teaching the principles of survival analysis, rather than a CONSORT-style flow diagram. A CONSORT-style flow diagram would be more informative, showing how many participants were initially assessed for eligibility, how exclusions were applied, and how the final analytical sample was derived.

 - In the Methods section, the authors mention the use of the Cox proportional hazards model but do not report testing the proportional hazards assumption. Furthermore, I could not locate any figures of Kaplan–Meier curves in the Results section, which raises concerns about the validity and transparency of the analysis.

 Given the extensive revision timeline—almost three years since the initial submission—and the apparent failure to sufficiently address or justify the lack of amendments in response to my previous feedback, I regret that I must recommend rejection of this manuscript.

Is the work clearly and accurately presented and does it cite the current literature?

Partly

If applicable, is the statistical analysis and its interpretation appropriate?

Partly

Are all the source data underlying the results available to ensure full reproducibility?

Partly

Is the study design appropriate and is the work technically sound?

Yes

Are the conclusions drawn adequately supported by the results?

Partly

Are sufficient details of methods and analysis provided to allow replication by others?

Partly

Reviewer Expertise:

Clinical epidemiology, pharmacoepidemiology, and social epidemiology

I confirm that I have read this submission and believe that I have an appropriate level of expertise to state that I do not consider it to be of an acceptable scientific standard, for reasons outlined above.

# Reviewer report: Reviewer response for version 1

**DOI:** 10.5256/f1000research.135337.r178384

**Published:** 2023-06-26

**Authors:** Nat Na-Ek

**Affiliations:** 1 Division of Social and Administration Pharmacy, Department of Pharmaceutical Care, School of Pharmaceutical Sciences, University of Phayao, Mueang Phayao District, Phayao, Thailand

Overall, this piece of work on the benefits of medical cannabis in improving the survival rate of combined hepatocellular cholangiocarcinoma (cHCC-CC) patients is interesting. However, several issues need further clarification and improvements.

**Major points:**

1. It is important to recheck the accuracy of the provided data ( https://doi.org/10.6084/m9.figshare.2010119). I noticed inconsistencies in the reported figures, such as the percentages of male and female patients and the number of patients receiving each treatment modality. Additionally, there was a coding issue with one patient receiving medical cannabis coded as 12 in the current treatment variable without an explanation. Addressing these inconsistencies is crucial for the reproducibility of the results.
2. The authors did not mention whether they performed a proportional hazards assumption test in the statistical analysis. Upon re-analysis, I found that the treatment variable violated this assumption, indicating that the hazard ratio was not constant at 0.28 across the entire follow-up time as reported. More importantly, the significant association between medical cannabis and survival rate was observed only in the early follow-up (3 months), not the whole study period.
  Therefore, it is important for the authors to conduct a re-analysis and introduce an interaction term between the treatment variable and time using the time-varying covariate (TVC) option in STATA. When reporting the hazard ratio, the authors should present it across the range of follow-up (e.g., within 10 months) to ensure validity and reliability. More details and examples can be found at Bellera  *et al. *(2010) ^1^.
3. Please avoid making causal claims in an observational study. For example, the sentence "the medical cannabis increased overall survival rates among cHCC-CC patients" (conclusion of the abstract) should be revised to "the medical cannabis was associated with improved overall survival rates among cHCC-CC patients." This distinction is vital because several alternative explanations (e.g., bias, errors, confounding, effect modification, reverse causality) could account for the significant findings. For instance, patients who received medical cannabis might have been more closely monitored by physicians, or they might have had unobserved or unmeasured characteristics (residual confounders) that affected their prognosis positively. Additionally, there may have been discrepancies in the quality of care across different settings.
  Therefore, the authors should refrain from assuming causality to avoid exaggerating the significance of their results. It would be appropriate to include a cautionary statement in the discussion section, such as "as this is an observational study, we cannot infer causality, and a randomised controlled trial is needed to establish the efficacy of medical cannabis in cancer patients."
4. Please provide justification and references for each covariate selected as adjusting factors in the analysis. Furthermore, clarify why certain continuous variables (e.g., age, disease duration after registration) were categorised instead of using them as continuous scales. Additionally, I recommend running the analysis with age as a quadratic term (age + age ^2^), as it was found to be significantly associated with death, suggesting a non-linear relationship between age and mortality. Therefore, using a quadratic term for each continuous variable would be more appropriate as it preserves important information ^2^.
5. Provide more details about the medical cannabis used in the study, such as product details, dosage form, dose, and administration. This information is crucial for generalising the findings to a clinical setting and enabling reproducibility. Additionally, clarify what the standard treatment was in the study and whether it was consistent across different settings.
6. In the discussion section, provide more information about the individuals who were lost to follow-up and discuss how their exclusion might have influenced the findings. Is it possible to determine whether these patients were still alive at the end of the study or if they died soon after dropping out?
7. Discuss the potential impact of differences in the quality of care across settings on the survival rate of patients in the study. It would be helpful to perform a subgroup analysis according to settings and utilise the strata option in the Cox model.
  Furthermore, consider conducting subgroup analyses based on other variables such as sex, age group, and current treatment to assess whether effect modification plays a role in the findings. Sensitivity and subgroup analyses are necessary to ensure the robustness of the findings, particularly in an observational study.
8. In the discussion section, compare the survival rates of the study, particularly in the standard treatment group, with previous works. If applicable, discuss the reasons for any differences observed. This will help strengthen the external validity of the study.

**Minor issues:**

1. Use "multivariable" instead of "multivariate" when discussing regression models. The term "multivariable" refers to adding explanatory variables (X) in the regression model, while "multivariate" implies examining various outcomes (Y) simultaneously ^3^.
2. Spell "proportional hazards regression" with an "s" in "hazards" since the term "proportional" implies the existence of at least two hazards.
3. Be aware of the term person-months as it is not a person per month, but it is rather the product of patients and their corresponding follow-up time. So, the unit of the incidence rate in your work should be written as “100 person-months” not “100 person/month”.
4. To improve clarity, consider splitting Table 1 into three separate tables. Table 1 should focus solely on the characteristics of included participants, allowing for inferential statistics (e.g., independent t-test, chi-squared test) to test the association between each characteristic and exposure status. Then, create Table 2 to present details of the outcome variable according to exposure status. Finally, present Table 3 as the main findings regarding the association between treatment and all-cause mortality, including both crude (unadjusted) and adjusted hazard ratios. Additionally, including a Kaplan-Meier plot with a risk table would aid in visualising the survival rates between patients receiving medical cannabis and those receiving standard treatment.
5. If possible, please discuss the potential biological mechanisms or underlying explanations of how medical cannabis can improve the survival rate of cHCC-CC patients.

Overall, addressing these major and minor points will greatly enhance the clarity, validity, and reproducibility of your study.

Is the work clearly and accurately presented and does it cite the current literature?

Partly

If applicable, is the statistical analysis and its interpretation appropriate?

Partly

Are all the source data underlying the results available to ensure full reproducibility?

Partly

Is the study design appropriate and is the work technically sound?

Yes

Are the conclusions drawn adequately supported by the results?

Partly

Are sufficient details of methods and analysis provided to allow replication by others?

Partly

Reviewer Expertise:

Clinical epidemiology, pharmacoepidemiology, and social epidemiology

I confirm that I have read this submission and believe that I have an appropriate level of expertise to confirm that it is of an acceptable scientific standard, however I have significant reservations, as outlined above.

## References

[1] : Variables with time-varying effects and the Cox model: some statistical concepts illustrated with a prognostic factor study in breast cancer. *BMC Med Res Methodol* .2010;10: 10.1186/1471-2288-10-20 20 10.1186/1471-2288-10-20 20233435 PMC2846954

[2] : Quantifying the impact of different approaches for handling continuous predictors on the performance of a prognostic model. *Stat Med* .2016;35(23) : 10.1002/sim.6986 4124-35 10.1002/sim.6986 27193918 PMC5026162

[3] : Multivariate or multivariable regression?. *Am J Public Health* .2013;103(1) : 10.2105/AJPH.2012.300897 39-40 10.2105/AJPH.2012.300897 23153131 PMC3518362

# Reviewer report: Reviewer response for version 1

**DOI:** 10.5256/f1000research.135337.r170664

**Published:** 2023-06-01

**Authors:** Selamat Budijitno

**Affiliations:** 1 Department of Surgery, Dr. Kariadi Hospital, Faculty of Medicine, Universitas Diponegoro, Semarang, Central Java, Indonesia

Based on the STROBE criteria, most of this research has fulfilled the criteria. In my opinion, there are several things that need to be improved so that this research is better:

- There is no sufficient detail of the methods, especially on the eligibility criteria of participants, and the method of follow up that provided to allow replication by others.
- ​​​​​​There are no very important data as a confounder, namely the pain scale/level of pain on the criteria when matching participants. 
- It ​​​​​​would be clearer if the authors can explain the relationship between pain levels, quality of life, and survival rates in biomolecular terms. Such as, for example, mutations in the NMDA receptor in chronic pain, which will produce P protein which can increase the risk of advanced metastasis, NMDA receptors stimulates the MAPK and CaMK pathways, leading to CREB activation in tumor cells. NMDAR-interacting proteins and the downstream signaling effectors display features in common between the neuronal and metastatic cancer processes, such as cell adhesion, migration, and survival.
- In the results of the cohort study, it would be better if the authors can explain in the report the numbers of individuals at each stage of study – e.g., numbers potentially eligible, examined for eligibility, confirmed eligible, included in the study, completing follow-up, and the reason of dropped out participant.
- Give reasons for nonparticipation/dropped out participant in each stage. Consider use of a flow diagram.

Thank you.

Is the work clearly and accurately presented and does it cite the current literature?

Yes

If applicable, is the statistical analysis and its interpretation appropriate?

Yes

Are all the source data underlying the results available to ensure full reproducibility?

Partly

Is the study design appropriate and is the work technically sound?

Yes

Are the conclusions drawn adequately supported by the results?

Yes

Are sufficient details of methods and analysis provided to allow replication by others?

Partly

Reviewer Expertise:

Surgical Oncology, biomolecular, Immunology, epidemiology

I confirm that I have read this submission and believe that I have an appropriate level of expertise to confirm that it is of an acceptable scientific standard, however I have significant reservations, as outlined above.

# Response

**Published:** 2023-06-26

**Authors:** Narisara Phansila

**Affiliations:** Mahasarakham University, Thailand

1. There is no sufficient detail of the methods, especially on the eligibility criteria of participants, and the method of follow up that provided to allow replication by others. 
**Ans:** *The survival rate matches the history of the first day of treatment in the clinic. Copy the symptoms and physical examination of the patient. Diagnosis, treatment, address, and telephone number from the medical records recorded by the treating physician from September 2, 2019, to October 31, 2020; follow up on the patient's last status until April 30, 2021. Check the status and date of the patient's death from the Cancer Unit's patient tracking database or from the death certificate of the patient Check the correctness of the information. and import data for analysis.*
** **
2. There are no very important data as a confounder, namely the pain scale/level of pain on the criteria when matching participants. 
**Ans:** *The evaluation was not assessed because secondary data were used to track only the six-month outcome, censor, or event to assess survival. Starting from admission to treat both types, the inclusion criteria were likely to be met for all patients aged 18 years and over.*
3. It would be clearer if the authors can explain the relationship between pain levels, quality of life, and survival rates in biomolecular terms. Such as, for example, mutations in the NMDA receptor in chronic pain, which will produce P protein which can increase the risk of advanced metastasis, NMDA receptors stimulates the MAPK and CaMK pathways, leading to CREB activation in tumor cells. NMDAR-interacting proteins and the downstream signaling effectors display features in common between the neuronal and metastatic cancer processes, such as cell adhesion, migration, and survival. 
**Ans:** *This study has not investigated a relationship; we only track the survival rate over time. and find factors that are general information, but we will be publishing again about the quality of life and survival.*
4. In the results of the cohort study, it would be better if the authors can explain in the report the numbers of individuals at each stage of study – e.g., numbers potentially eligible, examined for eligibility, confirmed eligible, included in the study, completing follow-up, and the reason of dropped out participant. 
**Ans:** *This study used secondary data based on the results of the diagnosis and the treatment system.*
5. Give reasons for nonparticipation/dropped out participant in each stage. Consider use of a flow diagram.
**Ans:**** *Due to the use of medical records, use the available information If death is specified in the system, there will be a death certificate. The lack of follow-up data will be used as a censor, but it can be used to calculate the survival rate by using survival statistics.*
